# A novel proteasome inhibitor acting in mitochondrial dysfunction, ER stress and ROS production

**DOI:** 10.1007/s10637-012-9871-1

**Published:** 2012-09-14

**Authors:** Durvanei Augusto Maria, Jean Gabriel de Souza, Katia L. P. Morais, Carolina Maria Berra, Hamilton de Campos Zampolli, Marilene Demasi, Simone Michaela Simons, Renata de Freitas Saito, Roger Chammas, Ana Marisa Chudzinski-Tavassi

**Affiliations:** 1 Laboratório de Bioquímica e Biofísica- Instituto Butantan, Av. Vital Brazil, 1500–CEP, 05503-900 São Paulo, SP Brazil; 2 Departamento de Bioquímica, Universidade Federal de São Paulo, São Paulo, SP Brazil; 3 Programa de Pós-Graduação Interunidades em Biotecnologia, USP, IPT, Instituto Butantan, São Paulo, SP Brazil; 4 Faculdade de Medicina da USP, LIM24-Laboratório de Oncologia Experimental, Universidade de São Paulo, São Paulo, SP Brazil

**Keywords:** Apoptosis, Bcl-2 family protein, Caspase, Reactive oxygen species, Proteasome, Endoplasmic reticulum stress, Amblyomin-X

## Abstract

In cancer-treatment, potentially therapeutic drugs trigger their effects through apoptotic mechanisms. Generally, cell response is manifested by Bcl-2 family protein regulation, the impairment of mitochondrial functions, and ROS production. Notwithstanding, several drugs operate through proteasome inhibition, which, by inducing the accumulation and aggregation of misfolded or unfolded proteins, can lead to endoplasmic reticulum (ER) stress. Accordingly, it was shown that Amblyomin-X, a Kunitz-type inhibitor identified in the transcriptome of the *Amblyomma cajennense* tick by ESTs sequence analysis of a cDNA library, obtained in recombinant protein form, induces apoptosis in murine renal adenocarcinoma (RENCA) cells by: inducing imbalance between pro- and anti-apoptotic Bcl-2 family proteins, dysfunction/mitochondrial damage, production of reactive oxygen species (ROS), caspase cascade activation, and proteasome inhibition, all ER-stress inductive. Moreover, there was no manifest action on normal mouse-fibroblast cells (NHI3T3), suggesting an Amblyomin-X tumor-cell selectivity. Taken together, these evidences indicate that Amblyomin-X could be a promising candidate for cancer therapy.

## Introduction

Apoptosis induction has been the subject of many studies, not only of therapeutic potential, but also for a better understanding of the cell mechanisms involved in various forms of biological response, such as aging [1–3]. The process itself comprises a highly conserved mechanism, which can be induced by a variety of physiological or pathological conditions, in which caspases and mitochondria play a crucial role [4]. Except for death-domain receptors, the molecular mechanisms by which the many different pro-apoptotic stimuli, such as irradiation, chemotherapeutics or growth factor depletion, signal and initiate mitochondrial changes, are currently not well understood. There is agreement that cytochrome c action has two important consequences: (a) caspase cascade activation by the interaction of cytochrome c with Apaf-1 and procaspase-9; and (b) inhibition of the mitochondrial electron transfer chain, thereby resulting in the reduction of oxidative phosphorylation, inducement of reactive oxygen species (ROS) production, and finally, impairment of cellular ATP production during secondary necrosis [5].

Although mitochondria are the major source of ROS, these can also be derived from several intracellular sources, including NAD(P)H oxidase, xanthine oxidase, uncoupled nitric oxide synthase and ER stress [6–8]. Apoptosis is also regulated by various ROS and nitrogen species (RNS) [8–10]. ROS, such as hydrogen peroxide (H_2_O_2_), superoxide radicals (O_2_^•-^) and hydroxyl radicals (^•^OH), by-products of normal oxygen metabolism, participate in normal cell functions, and act as intracellular signaling molecules in a series of biological processes [9–11]. Nevertheless, imbalance in ROS production may have a cytotoxic effect. Furthermore, subtle changes in the RNS production rate, as nitric oxide (NO), may critically impact cell homeostasis, thereby initiating a series of cellular signaling processes, including apoptosis [9, 12].

Bcl-2 and p53 proteins are among those involved in mitochondrial dysfunction and damage, as well as ROS production [13, 14]. The Bcl-2 proteins are located within the outer mitochondrial membrane, the nuclear envelope and the endoplasmic reticulum (ER) of cells. Whereas anti-apoptotic proteins stabilize the mitochondrial membrane, pro-apoptotic proteins induce the release of cytochrome c, thereby activating effector caspases, such as caspase-3 [15–17]. p53 proteins, known as the "guardians of the genome", play an important role in maintaining DNA integrity and apoptosis induction, by selectively removing damaged cells and protecting the organism against cancer development [18–20]. It is well known that p53 are involved in the regulation of the transcriptional levels of pro- and anti-apoptotic genes, death receptors, and those other factors involved in the different steps of the apoptotic pathway [14]. For example, this protein can directly activate Bax [21], or directly bind to Bcl-XL [22] and Bcl-2 proteins, in order to induce mitochondrial permeabilization, and thus release cytochrome c.

Certain chemotherapeutic drugs have been specifically designed to induce apoptosis through mitochondrial dysfunction and damage, and ROS production, thus activating cytotoxic action. Nonetheless, some of these, most notably proteasome inhibitors, induce cell death through the ER-stress mediated apoptotic pathway [23–25]. ER organelle is involved in the synthesis, structural modification and transport of proteins and lipids [26]. When newly synthesized proteins in ER do not present a correct folding, they are retro-translocated to the cytosol, where they are immediately directed to proteassomal degradation [27, 28]. This mechanism plays an important role in reducing the amount of misfolded or unfolded proteins in ER. The blockage of proteasome proteolytic activity by pharmacological inhibitors, such as bortezomib/PS-341, severely compromises the removal of proteins with impaired conformational structure, thereby leading to their subsequent accumulation inside the ER lumen, with the consequential overloading [29, 30]. When these acute or chronic structural and functional disturbances reach the point of compromising ER integrity, there is a specific stress response, which, by various mechanisms, activates anti-apoptotic, apoptotic cell, or block cell cycle progression [31]. Amongst others, ER stress involves (a) specific transcriptional and translational responses, such as, phosphorylation of the alpha subunit of the translation initiation factor eIF (eIF2α), (b) the activation of chaperone 78 kDa glucose-regulated protein (GRP78), as well as nuclear transcription factors of growth arrest and DNA damage (GADD153), (c) ER-specific caspase-12, and (d) calcium imbalance [32, 33].

Within this context, Amblyomin-X was recently shown to be a proteasomal inhibitor, selectively pro-apoptotic activity in tumor human cells (SK-Mel-28 and Mia-Paca-2) [34]. This molecule, obtained in recombinant protein form, is a Kunitz-type inhibitor (GenBank accession AAT68575) identified in the transcriptome of the *Amblyomma cajennense* tick by ESTs sequence analysis of a cDNA library [35]. Simultaneous studies of Murine Renal Adenocarcinoma cells (RENCA) have shown that Amblyomin-X is also capable of inducing apoptosis in this lineage [36]. In this report, it was demonstrated that the pro-apoptotic mechanisms used by Amblyomin-X to trigger effects in RENCA cells, include pro- and anti-apoptotic Bcl family protein activation, dysfunction/mitochondrial damage, ROS production, caspase-cascade activation and proteasome inhibition, all capable of inducing ER stress.

## Material and methods

### Amblyomin-X expression

The recombinant protein was prepared as described elsewhere [35].

### Cell Culture

RENCA cells were cultured in RPMI, supplemented with 10 % fetal bovine serum, 0.1 mM of nonessential amino acids, 1 mM of sodium pyruvate, 2 mM of L-glutamine, 100 mg/mL of streptomycin sulfate and 100 U/mL of penicillin G. The strain was kindly provided by Dr. Maria Helena Bellini Marumo (Institute of Energy and Nuclear Research—IPEN—in São Paulo, Brazil), wich in turn received from Dr. Isaiah J. Fidler (The University of Texas M. D. Anderson Cancer Center, Houston, TX)[36–38]. It is an epithelial renal cell carcinoma of conventional type, clear cell (ccRCC) from spontaneous tumor growth in renal cortical tissue of mice of the Balb/c tha was isolated in 1969 and established as a cell line in 1973[39]. The NIH3T3 mouse fibroblast cells [ATCC No. CRL-1658] were cultured in DMEM , supplemented with 10 % fetal bovine serum, 100 mg/mL of streptomycin sulfate and 100 U/mL of penicillin G. RENCA and NIH3T3 cells were kept at 37 °C in a 5 % CO_2_ atmosphere. The medium was changed after 12 h, and then every 3 days.

### Cell micromorphological analysis by scanning electron microscopy (SEM)

In order to facilitate protein penetration and precipitation, and ensure optimal preservation of their ultrastructures, RENCA and NIH3T3 cells were fixed with 3 % glutaraldehyde for 24 h. Subsequently, the samples were washed 5 times in a cacodylate buffer, prior to post-fixing in 4 % buffered OsO_4_ for 1 h, followed by further buffer-washing. Prior to treatment with or without 0.1 µM of Amblyomin-X, the cell suspension was centrifuged at 1500 rpm for 5 min, re-suspended in RPMI-1640 supplemented with 10 % FBS, and cultured in 2 cm^2^ petri dishes at a density of 10^6^ cells/mL. After 24-hours of treatment, the cells were referenced for normal NIH/3 T3 fibroblasts, before transferring to permeable critical-point apparatus for double dehydrating in alcohol baths at 30, 50, 70, 80, 95 and 100 % concentration. Several changes were required to so ensure complete removal of all the water content. Drying of the samples was performed at critical point apparatus using carbon dioxide and the plates received the metallic coating with gold by sputtering. Finished processing the material was observed and analyzed in a Philips XL30 scanning-electron microscope.

### Determination of the proteins involved in apoptosis and mitochondrial events by flow cytometry

Flow cytometry was applied to evaluating pro- and anti-apoptotic protein expression, cytochrome c measurement, the analysis of mitochondrial membrane potencial, and caspase activation. Accordingly, RENCA and NIH3T3 5x10^5^ cells were placed on 6-well-plates with a culture medium supplemented with fetal bovine serum. After cell adhesion, Amblyomin-X was added to the cell culture for 24 h, in a final concentration of 0.1 μM.

So as to assess Bcl-2 family expression and caspase 3 activation, cell concentration was first adjusted to 5x10^5^cells/mL, whereupon aliquots of 100 μL of cell suspension were permeabilized with a solution of Triton X-100 0.1 % in FACs buffer Flow (BD), and then incubated for 1 h at 4 °C with 1 μg of either of three specific antibodies, anti-Bad, anti-Bax or anti-Bcl-2, conjugated with FITC (Santa Cruz, USA), and 1 μg of specific antibody anti-Caspase 3 phosphorylated phycoerythrin conjugated-PE (Santa Cruz, USA), in either the presence or absence of its specific Ac-Asp-Glu-Val-sp-OH inhibitor (BioAgency Biotechnology). Subsequently, the cells were centrifuged for 10 min at 1500 rpm and washed with cold PBS. The supernatant was discarded, and the cell-button again suspended in PBS containing 1 % of paraformaldehyde. The reading and analysis of expression in arbitrary units of fluorescence of cell surface receptors were performed with a flow cytometer (Becton Dickinson, San Jose, CA, USA).

Cells present in the supernatant, as well as those trypsinized adherent, were transferred to cytometer tubes, and fixed with 4 % paraformaldehyde, in order to evaluate cytochrome c release and p53 expression. Cells were permeabilized with 0.5 % saponin in PBS for 30 min at room temperature. In sequence, cells were washed with a buffer Fac's flow containing 0.1 % saponin in PBS solution (BSA/saponin), and then incubated with monoclonal antibody anti-cytochrome c (Santa Cruz USA) and anti-p53 (Santa Cruz, USA), at a concentration of 1 μg of antibody per 10^6^ cells, during 18 h at 4 °C. They were then washed with BSA/saponin and incubated with a secondary antibody conjugated with phycoerythrin—PE (1 μg). After incubation for 2 h at 37 °C, they were again washed with PBS and fixed in 1 % paraformaldehyde. Accompaniment throughout the whole process was done by isotype control.

Electric potential was assessed by mitochondrial effluxing of fluorescent probe Rhodamine-123, with a functional MDR phenotype as control. Briefly, 300 μL of cell suspension were incubated with or without Rhodamine-123 probe (200 ng/mL) in a humidified 5 % CO2 incubator for 45 min at 37 °C. After centrifugation for 30 min at 1500 rpm, the cells were washed in cold PBS, and mitochondrial potential measured with a flow cytometer. Histograms of fluorescence intensity were obtained from the gates of cells tested. The results were analyzed as mean fluorescence intensity (MFI), by MFI ratio of the sample under study and its negative control. In this study, 1x10^4^ cells per sample were analyzed with Cell Quest (BD) version 3.1, and FSC (size) and SSC (granularity/complexity) FL1/FL2 as parameters, to evaluate fluorescence intensity of the binding reaction antigen/antibody conjugated to PE and/or FITC. All acquisitions were done in three independent experiments.

### Proteasome activity

Proteassome activity was determined according to Chudzinski et al. [34]. RENCA and NIH3T3 cells were treated with 0.1 μM of Amblyomin-X for two periods of 4 and 16 h. The cells were then washed twice with PBS and mechanically lysed. After quantification of total proteins in the cell extract, aliquots of 15–30 μg were separated for measuring proteasome activity. Fluorogenic peptides (AMC, 7-amide-4-methylcoumarin; Calbiochem, San Diego, CA, USA) were used to determine proteasomal activity, Suc-LLVY-AMC, the standard peptide for assessing chymotrypsin-like activity and z-ARR-AMC, was used to determine trypsin-like activity. The cell extracts obtained were incubated at 37 °C with 125–500 mM of fluoropeptides. Fluorescence emission at 440 nm (excitation at 365 nm) was recorded for 1 h. The calculation of final results, expressed as the percentage of hydrolysis measured in total extracts from untreated control cells, was based on the difference between total cell-extract hydrolytic activity, and the activity determined in samples previously incubated with standard proteasome inhibitors (lactacystin or NLVS). All the experiments were carried out in triplicate.

### Calcium measurement by confocal microscopy

Before measuring changes in [Ca^2+^]_*i*_ levels in RENCA cells by calcium imaging with a LSM 510 Meta confocal microscope (Zeiss, Jena, Germany), 2.5x10^5^ cells were seeded and attached to cover slides in p60-mm culture dishes (Nalge Nunc International, Rochester, NY), prior to pre-treatment with 0.5 μM of Amblyomin-X at intervals of 5 and 24 h. After loading for 30 min at 37 °C with 4 μM Fluo-3 AM (Sigma Chemical, St. Louis, MO) in the presence of 0.5 % Me2SO and 0.1 % of the nonionic surfactant pluronic acid F-127, the cells were then washed three times with DMEM containing 10 % FBS. Fluo-3 AM fluorescence excitation was done by Argon ion laser at 488 nm, with fluorescence-emission collection at 510–530 nm with a band-pass filter. The changes in [Ca^2+^]_*i*_ levels were recorded every 10 s. After reading each sample, a 4-Br-A23187 ionophore (5 μM) and an EGTA chelating compound (10 mM) were used to determine maximal (Fmax) and minimal (Fmin) fluorescence values, respectively. Intracellular free-calcium concentrations were calculated from relative fluorescence values by using the equation [Ca^2+^]_*i*_ = Kd (F – Fmin)/(Fmax – F), assuming 450 nM Kd for fluo-3 calcium binding [40].

### Gene expression analysis related to ER stress response by real-time PCR

Polymerase chain reaction (PCR) was applied to real time quantitative expression analysis of those genes related to ER-stress response. cDNA strands were constructed with total RNA extracted from RENCA cells, using a cDNA Cycle Kit for RT-PCR kit (Invitrogen^™^, Life Technologies Inc.), according to manufacturer's instructions. Specific primers were used in the formation of double-stranded cDNA. The StepOne Plus Real-Time PCR System (Applied Biosystems, Foster City, CA, USA) was applied to cDNA amplification. Specific double-tranded DNA was labeled with SYBR Green I provided in the SYBR Green PCR Master Mix (Invitrogen^™^, Life Technologies Inc.), to so facilitate quantitative detection of PCR products in a reaction volume of 25 μL. The PCR procedure was an initial 10 min at 95 °C, followed by 40 cycles of denaturation of 15 s at 95 °C, 30 s at 60 °C, and finally 30 s at 72 °C. The GAPDH (Glyceraldehyde-3-phosphate dehydrogenase) gene expression level was used to normalize differences in RNA isolation and degradation. The results were analyzed by relative quantification between groups: control, using 2^-ΔΔCT^ [41].

### Determination of GRP78 and GADD153 protein expression by Western blot

RENCA cells were grown in an appropriate medium until reaching 80 % confluence. Once stabilized, the culture medium was replaced, whereupon the cells were stimulated with 0.5 μM of Amblyomin-X for periods of 2, 6 or 24 h. They were then lysed with a RIPA buffer (1 % deoxycholate, 150 mM of NaCl, 1 % SDS, 10 mM of NaF, 1 % Triton X-100, 50 mM of Tris–HCl), together with the protease inhibitors, aprotinin (2 mg/mL), fenilmetilsulfonilfluoreto (PMSF) (1 mM), and orthovanadate phosphatase (1 mM). The resultant products were then incubated on ice for 20 min. After protein quantitation by the bicinchoninico acid (BCA) method, using BSA as standard protein curve, 30 μg from each sample underwent electrophoresis on 10 % SDS gel, prior to transferring to a PVDF membrane (Hydrophobic polyviniylidene difluoride, Healthcare, USA), using as transfer buffer 0.25 M Tris, 0.19 M glycine and 20 % methanol, under a constant voltage of 100 V for 1 h at 4 °C. In order to block nonspecific sites, the membrane was incubated three times, first with a solution of 5 % BSA in TBS-Tween (20 mM of Tris–HCl pH 7.4, 0.15 M of NaCl and 0.2 % Tween), then with the primary antibodies anti-GRP78 1:1000 (Santa Cruz Biotechnology Inc, CA, USA) and anti-GADD153 1:200 (Santa Cruz Biotechnology Inc, CA, USA), using as internal control anti-GAPDH 1:5000 (Sigma Aldrich, MO, USA), and finally with an HRP-conjugated secondary antibody (Sigma-Aldrich, MO, USA). Immunostaining was revealed with an ECL solution and detected by LAS4000 ImageQuant equipment (EG, Germany). An Amersham Rainbow Full-Range molecular weight marker (GE Healthcare, USA), was used for comparing the position of the bands on the gel in use. The ImageJ analysis program (National Institute of Health, USA) was used for quantification of the bands thus found.

### Determination of NO and H_2_O_2_ production

In order to determine NO production, 1x10^5^ RENCA cells were seeded onto 96-well-plates for posterior treatment with 0.1 μM of Amblyomin-X during 48 h. NO production was monitored in the extracellular medium by assessing nitrite levels with a Griess reagent. Briefly, 50 μL of cell-free supernatants were added to 100 μL of Griess reagent (5 % H3PO4, 1 % sulfanilamide, 0.1 % N-1-naphtyl-ethylendiamine). Absorbance was measured at 540 nm. Various concentrations of sodium nitrite were used in the construction of a standard curve. All biochemical analyses were performed in triplicate.

The assessment of H_2_O_**2**_ levels by microassaying, using a technique described and adapted by Pick and Mizel [42], consisted of the following. 2x10^5^cells/mL of adherent RENCA cells was suspended in 1 mL of RPMI-1640. 100 μL of the so formed suspension were placed into flat-bottomed 96-well culture plates. In order to induce cell adhesion, the plates were incubated for 1 h at 37°C in a humidified atmosphere (5 % CO_2_). The wells were then washed with PBS to remove non-adherent cells. A phenol red solution was then added, prior to H_2_O_**2**_ assaying. This was followed by incubation at 37 °C in a humid atmosphere for 1 h. The reaction was stopped by adding 1 N sodium hydroxide (NaOH). The tests were done in quadruplicate. Control samples, without PMA stimulation, were simultaneously evaluated to assess the spontaneous production of H_2_O_**2**_. Absorbance was determined by way of an ELISA reader with a 620 nm filter, to thus obtain optical density (OD). The results are presented in nmols of H_2_O_**2**_.

### Statistical analysis

For comparative analysis of the two groups, treated or untreated with Amblyomin-X, statistical significance was determined by one- or two-way variance analysis (ANOVA) followed by Bofferoni correction, as well as the Tukey-Kramer or Dunnett post-tests**,** using GraphPad Prism 5.0 software (GraphPad Software Inc., San Diego, CA). The criteria for statistical significance, set at *P* < 0.05, were represented by an asterisk (*). Data are expressed as mean ± SEM.

## Results

### Morphological apoptosis characteristics were observed in RENCA cells treated with Amblyomin-X

**Fig. 1:**
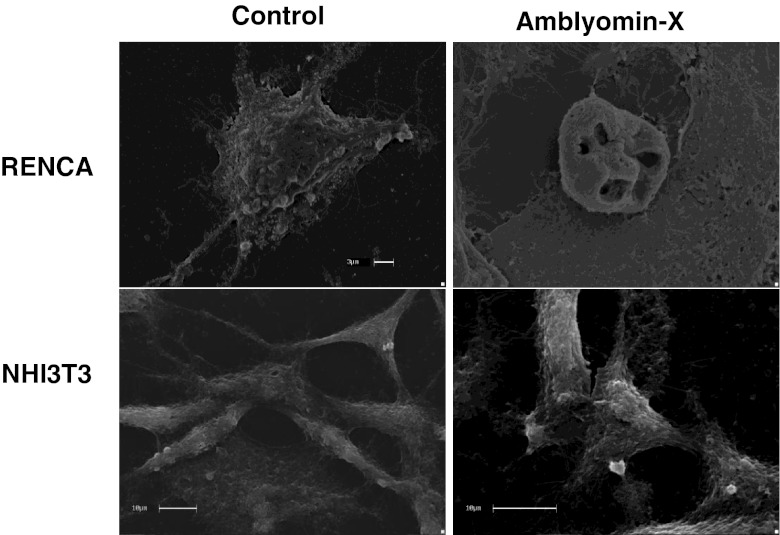
Morphological differences between RENCA and NIH3T3 cells treated with Amblyomin-X. Photomicrographs of cells treated with 0.1 μM of Amblyomin-X for 24 h, and untreated ones (control), observed and analyzed by SEM. **(RENCA Control)** Details of extracellular matrix organization. **(RENCA Amblyomin-X)** Apoptotic bodies and cell-membrane disruption. **(NHI3T3 Control)** Spindle cells, extracellular matrix organization and fibrillar collagen arrangement. **(NHI3T3 Amblyomin-X)** Fibroblastoid cells adhered to the surface and extracellular matrix organization

RENCA cells treated with Amblyomin-X presented morphological differences when compared to those un-treated (Fig. 1). In the control group, a novel cell structure was formed, through the expansion of a central condensed part into long, branched structures projecting from cytoplasmic interconnections and expansions of the cytoplasm and the cytoskeleton (Fig. 1). In contrast, the group treated with Amblyomin-X presented striking morphological changes, characteristic of cells undergoing apoptosis, with apoptotic body formation, the reduction of microvilli, loss of adhesion to the matrix, and formation of membrane blebs. There is no organization of the extracellular matrices, but appreciable morphological changes, such as the formation of round cells and cell debris (Fig. 1). Interestingly, NIH3T3 cells in both the control and treated groups showed the same pattern of cell morphology, with the presence of fibroblastoid spindle cells, adhered to the contact surface of the culture plate, and the formation of three-dimensional fiber and fibril high-density networks connecting the external surfaces of cells with cytoplasmic projections to adjacent cells. Furthermore, there were no morphological modifications, such as cell rounding or debris, and the well-preserved extracellular matrix was juxtaposed between firmly adhered cells and those recently proliferated. Moreover, cell division was clearly visible (Fig. 1).

### Amblyomin-X affects the balance between pro-apoptotic and anti-apoptotic proteins and mitochondrial integrity

**Fig. 2:**
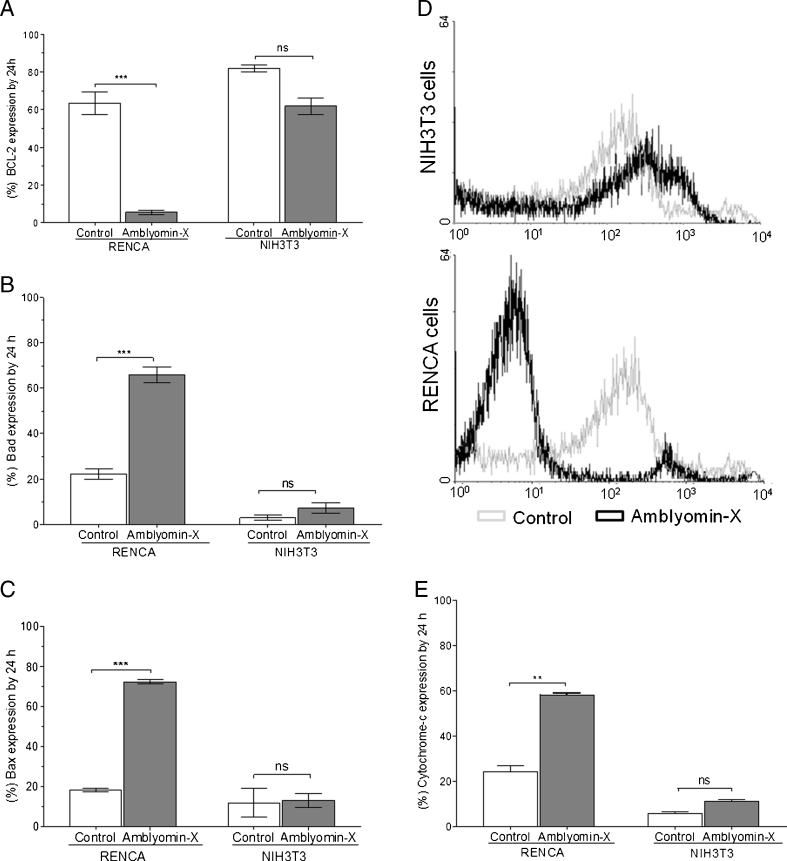
Imbalance between pro-and anti-apoptotic Bcl-2 family proteins, accompanied by mitochondrial function impairment, is caused by Amblyomin-X. RENCA and NHI3T3 cells were treated with 0.1 μM of Amblyomin-X during 24 h. **a** Bcl-2 expression. **b** Bad expression. **c** Bax expression. **d** Representative histograms of mitochondrial membrane potential. **e** Release of cytochrome c. Experiment-monitoring was by flow cytometry. The data are presented as mean ± SEM. *P < 0.05 vs. control

**Fig. 3:**
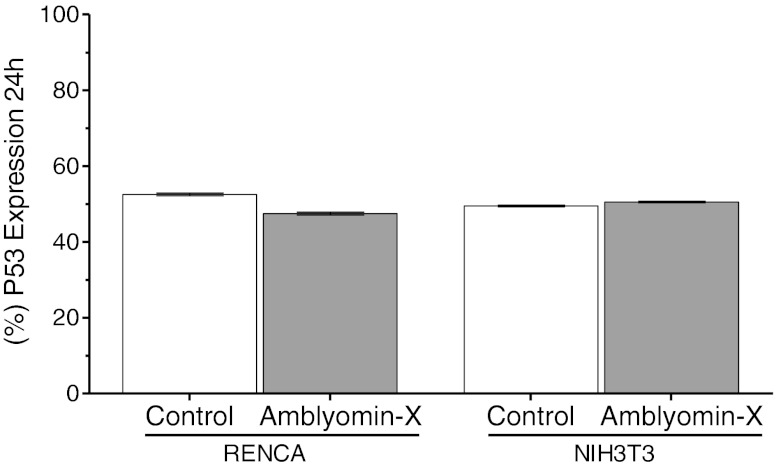
Amblyomin-X action is p53 independent. RENCA and NHI3T3 cells were treated with 0.1 μM of Amblyomin-X during 24 h. Experiment-monitoring was performed by flow cytometry. The data are presented as mean ± SEM. *P < 0.05 vs. control

Analysis of Bcl-2 family expression levels by flow cytometry revealed that treatment with 0.1 μM of Amblyomin-X induces imbalance between pro- and anti-apoptotic proteins in RENCA, but not in NHI3T3 cells. After 24 h of Amblyomin-X treatment, there was a significant change in protein expression levels, with an approximate 6-fold reduction in anti-apoptotic Bcl-2 proteins, and a 3.5-fold increase in Bax and Bad (Fig. 2a, b and c). However, no changes were observed in p53 expression levels in either RENCA or NIH3T3 cells (Fig. 3), an indication that this protein is not involved in the regulation of either expression or cytoplasmic activation in the Bcl-2 family.

As these proteins play a role in the maintenance of mitochondrial integrity, and since dysfunction has been noted, it was decided to investigate whether changes in Bcl-2 family expression levels were accompanied by changes in mitochondrial potential. Thus, by using the same concentration of Amblyomin-X and the same treatment period, it was observed that Amblyomin-X was capable of significantly reducing the mitochondrial potential in both adherent RENCA cells and those present in the supernatant, with no changes in NHI3T3 cells (Fig. 2d). Contributing to this result, there was an approximate 3-fold increase in the release of cytochrome c into the cytosol in RENCA cells treated with Amblyomin-X, when compared to control group. This was not observed in NHI3T3 cells, where no changes in cytochrome c release were observed (Fig. 2e).

### The effect of Amblyomin-X on caspase 3 catalytic activity

**Fig. 4:**
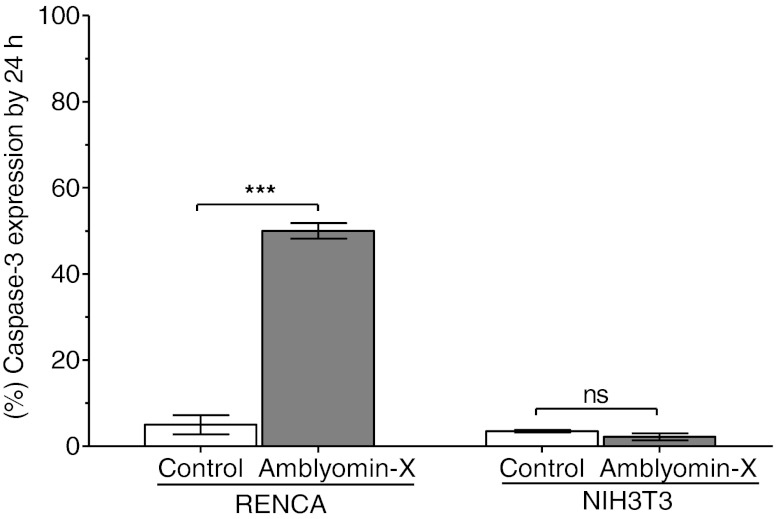
Amblyomin-X induces caspase 3 activation. Detection of phosphorylated caspase-3 by flow cytometry in RENCA and NHI3T3 cells treated with 0.1 μM of Amblyomin-X during 24 h. The data are presented as mean ± SEM. ***P < 0.0005 vs. control

Caspase 3 catalytic activity in RENCA and NHI3T3 cells was evaluated after a 24-hour-treatment with 0.1 μM of Amblyomin-X. The proportion of caspase 3 phosphorylation and activation by Amblyomin-X in RENCA cells was about 7 times higher than in the control group (Fig. 4). This could arise from the observed cytochrome c release (Fig. 2e). On the other hand, no like difference was observed in either treated or untreated NHI3T3 cells (Fig. 4).

### Proteasome inhibition by Amblyomin-X

**Fig. 5:**
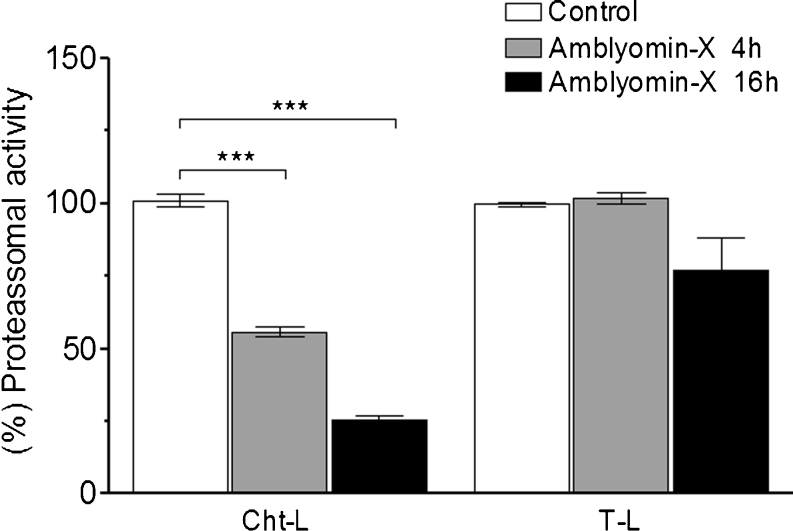
Inhibition of proteasome activity by Amblyomin-X. RENCA cells were treated with 0.1 μM Amblyomin-X for 4 and 16 h, as indicated. ChT-L and T-L proteasomal activities were defined in the cell extracts, as described in Material and Methods. Results shown are expressed as percentages of control samples. ChT-L, chymotrypsin-like and T-L, trypsin-like activities, respectively. The data are represented as mean ± SEM. ***P < 0.0005 vs. control

Previous studies have shown that Amblyomin-X is capable of negatively modulating proteasome activity in SK-Mel-28 and Mia-Paca-2 tumor human strains, through preferentially inhibiting the trypsin-like site and increasing the pool of poly-ubiquitinated proteins [34]. This strongly implies that this activity could be an Amblyomin-X target for triggering cytotoxic effects. Results obtained with RENCA cells showed that 4 and 16 h after treatment with 0.1 μM Amblyomin-X, there was a significant decrease in chymotrypsin-like proteasome activity (Fig. 5). After 16 h of incubation, the reduction was 4-fold, when compared to controls.

### Amblyomin-X-induces ER stress in RENCA cells

On seeking to correlate the effects of Amblyomin-X on proteasome functions and ER stress in RENCA cells, it was decided to evaluate whether this molecule was capable of causing ER stress. Hence, its capacity to interfer with [Ca^2+^] and the expression of targets related to ER stress, was analyzed.

**Fig. 6:**
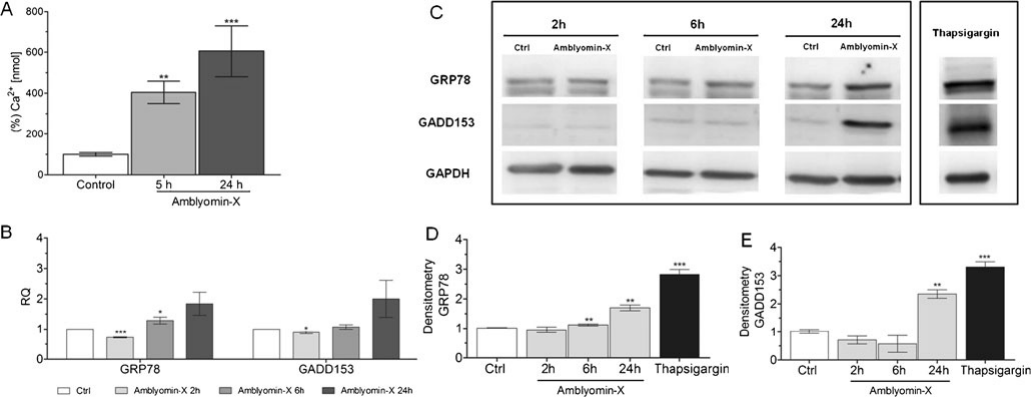
Amblyomin-X induces ER stress in RENCA cells. **a** Through confocal microscopy—the graphical representation of Ca^2 +^ quantitative influx in unstimulated cells, and RENCA cells stimulated with 0.5 μM Amblyomin-X for 5 and 24 h. **b** By real-time PCR—levels of gene expression of targets related to ER stress. Cells were treated with 0.5 μM of Amblyomin-X for 2, 6 and 24 h. **c** Western blot analysis of samples with or without treatment (Ctrl) using anti-GRP78, anti-GADD153 and anti-GAPDH, as endogenous controls. **d** and **e** Densitometry analysis of protein bands revealed with anti-GRP78 and anti-GADD153 relative to control. Values are mean ± SD of three independent experiments. *P < 0.05 compared to control (untreated cells)

Through microfluorimetry, the increase in [Ca^2+^]_*i*_ was instantaneous, although not transient, in Amblyomin-X treated RENCA cells, suggesting an induced calcium homeostasis disturbance. For instance, immediately after applying of 0.1 μM of Amblyomin-X, there was a change in the pattern of fluorescence, without the peak response reaching a maximum (data not shown). Thus, in order to assess [Ca^2+^]_*i*_ mobilization after longer periods of treatment, quantification by confocal microscopy was resorted to, whereby it was possible to visualize the fluorescence of cells pre-incubated with Amblyomin-X and treated with Fluo-3 AM (Fig. 6a). The analysis of [Ca^2+^]_*i*_ elevation during Amblyomin-X treatment, revealed that after 5 and 24 h, there was a significant increase—about three- and five-fold, respectively—in the amount of free ions, when compared to control. Nevertheless, on comparing 5-hour and 24-hour treatments, there was no significant difference indicating that Amblyomin-X had reached its maximum response.

The capacity of Amblyomin-X to affect gene expression of targets related to ER stress pathways was tested through real-time PCR. Interestingly, after treating RENCA cells with 0.5 μM of Amblyomin-X for 2 h, a significant reduction of around 30 % in mRNA GRP78 levels occurred, followed by a pronounced increase in expression after 6 and 24 h, of 30 and 80 %, respectively (Fig. 6b). A similar pattern was observed for the GADD153 gene expression, with a 12 % reduction in expression after 2 h of treatment, and an increase of about 15 % and 100 % after 6 and 24 h, respectively, without any significant difference (Fig. 6b).

It was thus indicated that ER is involved in the mechanism of Amblyomin-X action, although it was not clear whether this was through GRP78 reduction or ER stress. Thus, it was decided to assess the levels of GRP78 and GADD153 protein expression under the same conditions of real-time PCR. A correlation between gene and protein expression became apparent. After 24 h of treatment with Amblyomin-X, there was a 70 % increase in GRP78 protein levels, compared to untreated cells (Fig. 6c and d). Moreover, although GADD153 protein levels decreased in the interval between 2 to 6 h, this was followed by a significant increase of about 235 % in treated cells after 24 h (Fig. 6c and e). Thapsigargin was used for 6 h, as positive control at 0.5 μM.

### Modulation of NO and H_2_O_2_ production by Amblyomin-X

**Fig. 7:**
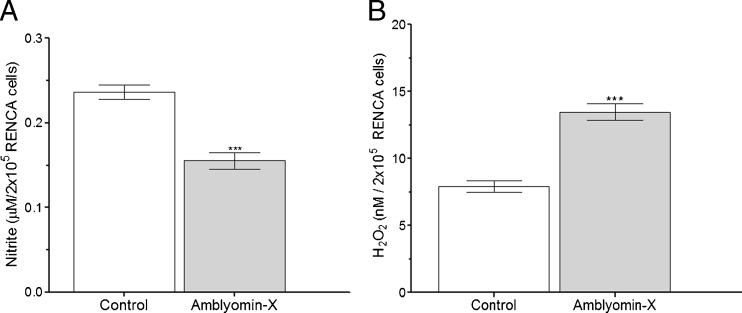
Changes in oxidative conditions of RENCA cells in the presence of Amblyomin-X. **a** NO levels in RENCA cells treated with 0.1 μM of Amblyomin-X for 48 h. **b** H_2_O_2_ levels in RENCA cells treated with 150 nM of Amblyomin-X for 24 h. The values are the mean ± standard deviation of three independent experiments. *P < 0.05 vs. control

The production of NO and H_2_O_2_ in RENCA cells was analyzed along the same lines followed in the experiments with Ca^2+^ and the expression of targets related to ER stress. After 48 h of 0.1 μM Amblyomin-X treatment, there was a reduction of 40 % in NO production, when compared to untreated cells (Fig. 7a), and after 24 h, a 44 % increase in H_2_O_2_ levels (Fig. 7b), thereby indicating that changes in oxidative conditions of RENCA cells treated with Amblyomin-X induced ROS production.

## Discussion

Cell death and survival are topics under constant discussion in scientific literature, however, it has become increasingly evident that these processes are regulated by a sophisticated balance between pro- and anti-apoptotic proteins and highly dynamic interactions that lead cells to a final destination [43, 44]. Hence, the present study demonstrated that Amblyomin-X promotes apoptotic morphological characteristics into tumor cells, accompanied by imbalance between the protein levels of Bcl-2 family members by decreasing anti-apoptotic and increasing pro-apoptotic levels, without p53 participation. Interestingly, there are several clinical and preclinical anti-tumor drugs, for example, ABT-737, that target members of the Bcl-2 family, and many studies argue that the reduction of Bcl-2 and Bcl-XL would be sufficient to induce cell apoptosis [13, 45, 46].

Mitochondria are also predominant target in the search for novel anti-tumor therapies. Accordingly, interest has recently been focused on a new class of drugs, the mitocans, which act through tumor cell mitochondria, without affecting normal ones [47, 48]. The mechanism of their action is based on the ability to disrupt cellular energy production systems by way of mitochondria, thereby leading to an increase in reactive oxygen species and the activation of cell death by signaling mitochondria dependent pathways. Apart from their efficacy, these drugs have become additionally attractive through presenting few side effects on normal cells in in vivo experiments [48]. These characteristics were observed in tumor cells treated with Amblyomin-X, by compromising the mitochondrial membrane potential, the release of cytochrome c, and the increased production of ROS, observed by an increase in H_2_O_2_ levels. These results suggest, thereby, that these responses can lead to caspase 3 activation, ultimately culminating in apoptosis.

Apparently, Amblyomin-X induces ER stress by negatively modulating proteasome activity. This is characterized by an increase in the concentration of free intracellular Ca^2+^ ([Ca^2+^]_*i*_), and changes in GRP78 and GADD153 protein levels, considered classical markers of the process. Proteasome inhibition can also lead to the accumulation of proteins bearing conformational-structure damage, a condition which can lead to ER stress [29, 30, 49]. Cell response consists of the release of ER Ca^2+^ stores, which is either taken up by mitochondria, or directed to other intracellular destinations, thereby activating a signaling cytoprotective response, denominated ‘unfolded protein response’ (UPR) [50]. One of the characteristic effects of UPR is translational attenuation, a form of reducing new-protein synthesis, and so avoiding the accumulation of proteins with conformational defects. This is accomplished by phosphorylation of the eIF2α, an event that redirects transcript alternative targets, such as chaperones (e.g., GRP78), and proteasome subunits [32]. However, if UPR is suppressed by sustained proteasome inhibition, stress can lead to severe and prolonged ER-activating death pathways [25, 51]. In parallel, the induction of transcription factors, such as pro-apoptotic growth arrest and DNA gene product GADD153, may also occur. GADD153 is a nuclear transcription factor that represses Bcl-2 promotors, and is capable of sensitizing mitochondria to the pro-apoptotic effects of BH3-only proteins. Its over-expression can lead to cell-cycle arrest and apoptosis [52]. GADD153 expression, mainly regulated at the transcriptional level, is one of the most highly induced during ER stress [52].

There appear to be several anti-tumors, such as curcumin, salubrinal, bortezomib and sorafenib, whose individual action are similar of Amblyomin-X mechanism, by inhibiting proteasome activity and causing ER stress [23, 24, 29, 53]. Similarly, some of these drugs act selectively on tumor cells. There is some discussion to explain this phenomenon, i.e., cycling cells are possibly more sensitive to proteasome inhibition than resting ones. This is probably due to so many crucial cell-cycle checkpoints being regulated by proteasome activity, or by displaying a higher baseline rate of translation compared to other cells. This could make them more vulnerable to protein buildup, and the subsequent ER stress caused by proteasome activity. Even so, as yet, nothing is conclusive [23, 24, 29, 53].

**Fig. 8:**
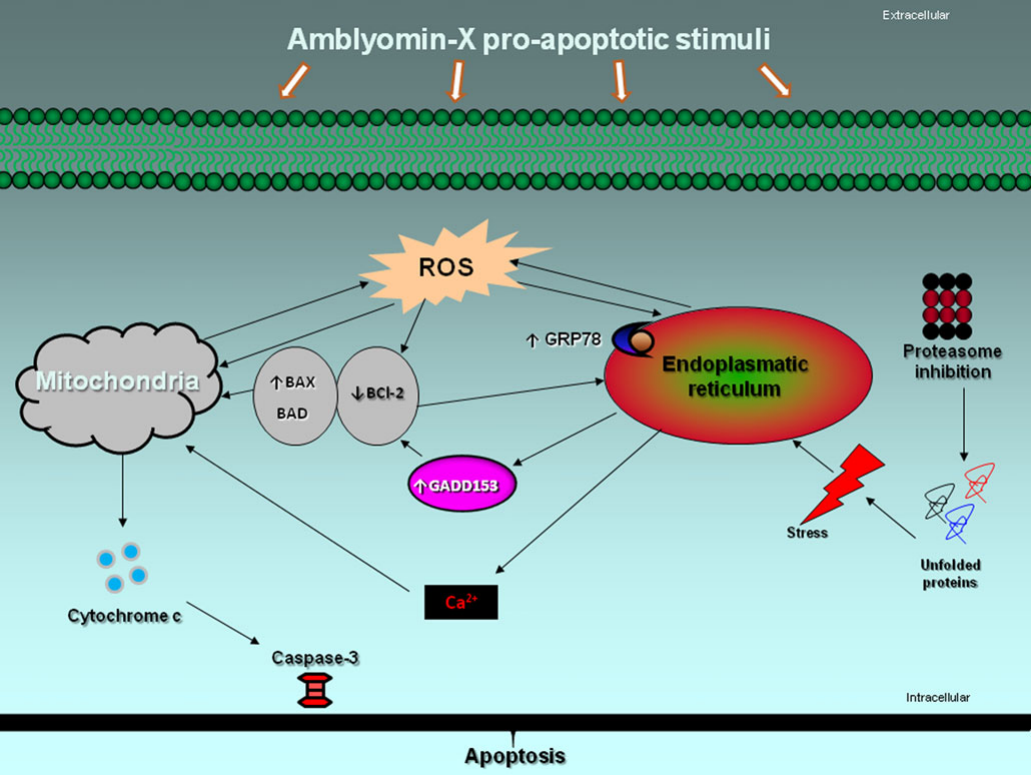
Possible mechanisms involved in Amblyomin-X pro-apoptotic action in RENCA cells. Amblyomin-X causes: (i) protein imbalance between pro- and anti-apoptotic Bcl-2 family proteins; (ii) mitochondrial dysfunction, marked by changes in its membrane potential and release cytochrome c; (iii) activation of caspases, (iv) proteassomal inhibition; (v) ER stress, characterized by induced expression in the respective protein markers (GRP78 and GADD153) and changes in (Ca^2+^) levels. Hypothetically, Amblyomin-X could act separately in these cell pathways responses, or even synergistically between them, converging in apoptosis. Nevertheless, more conclusive studies are required to confirm this hypothesis (adapted from [9, 33, 54–57])

Apparently, there is an interrelationship between mitochondrial dysfunction, ROS production, and ER stress. Mitochondrial dysfunction and ER stress are capable of producing ROSs, or even inducing the establishment of such cell conditions [54]. ER stress and ROS production also play a role in the modulation of Bcl-2 family proteins, as well as inducing mitochondrial damage [9, 55]. For example, as shown here, the Amblyomin-X induced increase in H_2_O_2_ and decrease in NO levels are both related to Bcl-2 protein instability and deactivation. Concomitantly, the Bcl-2 pro-apoptotic protein family can initiate apoptosis via ER, thereby directly affecting Ca^2 +^ stocks and caspase-12 activation [54–57]. It was found that proteasome inhibition (4 and 16 h of treatment) and responses related to ER stress, such as changes in gene expression of ER stress marker proteins and (Ca^2 +^)_*i*_ levels, were observed earlier (between 5 and 6 h of treatment), than responses related to mitochondrial damage, such as, cytochrome c release and ROS production (24 h of treatment). This presupposes Amblyomin-X begins pro-apoptotic signaling by way of ER stress. Notwithstanding, there is no way of ruling out the possibility of this molecule activating these pathways separately, and that their individual cellular response converge in apoptosis, or proceed until reaching the point of synergy between the pathways (Fig. 8). Novel experimental approaches are required to confirm this hypothesis.

## Conclusions

Amblyomin-X is an FXa inhibitor of the Kunitz-type that has anti-tumor activity. Even so, the cell-events triggered by this molecule have not, as yet, been finally defined. Nevertheless, its apoptotic effect on RENCA cells is hereby confirmed. This effect comprises: (a) induces protein imbalance between pro-and anti-apoptotic Bcl-2 family proteins; (b) mitochondrial dysfunction, marked by changes in its membrane potential and release of cytochrome c; (c) caspase 3 activation, (d) proteasomal inhibition; (e) ER stress, characterized by the induction of specific protein marker expression (GRP78 and GADD153) and changes in (Ca^2+^)_*i*_ levels (Fig. 8). In view of its selective action on tumor cells, since no significant change was observed in NIH3T3 cells treated with Amblyomin-X, and of the current efforts to develop effective cancer-combat therapies, these results are relevant to understanding the Amblyomin-X mechanism of action, and to its appointment as an effective therapeutic agent candidate.

## Abbreviations

ROS: reactive oxygen species
RNS: reactive nitrogen species
NO: nitric oxide
ER: endoplasmatic reticulum
eIF2α: alpha subunit of translation initiation factor
GRP78: 78kDa glucose-regulated protein
GADD153: nuclear transcription factors of growth arrest and DNA damage
RENCA: murine renal adenocarcinoma
SEM: scanning electron microscopy
UPR: unfolded protein response
[Ca^2+^]_*i*_: intracellular calcium concentration
